# Anti-Fibrotic Properties of a Decellularized Extracellular Matrix Scaffold from Porcine Small Intestinal Submucosa in Normal Human and Keloid Fibroblasts

**DOI:** 10.3390/ijms262411764

**Published:** 2025-12-05

**Authors:** Pradipta Banerjee, Rae Ritchie, Grace Lander, Amitava Das, Michael Hiles, Gayle Gordillo, Chandan K. Sen, Sashwati Roy

**Affiliations:** 1 McGowan Institute for Regenerative Medicine (MIRM), Department of Surgery, School of Medicine, University of Pittsburgh, Pittsburgh, PA 15213, USA; prb112@pitt.edu (P.B.); gmg112@pitt.edu (G.G.); c.k.sen@pitt.edu (C.K.S.); 2 Indiana Centre for Regenerative Medicine and Engineering (ICRME), Indiana University Health Comprehensive Wound Centre, School of Medicine, Indiana University, Indianapolis, IN 46202, USA; glander@iu.edu (G.L.); chemitava@gmail.com (A.D.); 3 Cook Biotech Inc., West Lafayette, IN 47906, USA; rritchie@rtix.com (R.R.); rolling.mike@gmail.com (M.H.)

**Keywords:** extracellular matrix, keloid fibroblast, thrombospondin-1, and fibronectin-1

## Abstract

Uncontrolled fibrosis via excess deposition of extracellular matrix (ECM) is a hallmark of hypertrophic scars and keloids. A decellularized ECM biomaterial from porcine small intestinal submucosa (SIS; Biodesign or BioD, Cook Biotech, Inc.) is widely used in clinical applications for tissue repair. The objective of the current study was to test the effects of BioD scaffolds, as compared with collagen constructs, on normal human skin (nFB) and keloid fibroblasts (kFBs). Immortalized human dermal fibroblasts (hFBs) and human keloid fibroblasts (hKFs) were utilized for all experiments. Cells were cultured either on BioD membranes or on collagen gel (used as a control). To investigate pro-fibrotic signaling pathways, real-time quantitative PCR (qPCR), ELISA, and gene knockdown studies were conducted on cultured cells. ECM gene expression array revealed that BioD significantly attenuated (*p* < 0.05) the expression of thrombospondin-1 and fibronectin-1, two drivers of fibrosis in nFB as well as kFB. BioD-repressed thrombospondin-1 and fibronectin-1 gene expression manifested as significant downregulation (*n* = 5–6; *p* < 0.05) of both proteins in nFB and kFB. The levels of latent transforming-growth factor (LAP-TGFβ-1) were markedly reduced (*n* = 5; *p* < 0.05) in both nFB and kFB cultured on BioD, but not the other constructs. Knockdown of FN1 using siRNA significantly attenuated (*n* = 5, *p* < 0.05) pro-fibrotic responses, including expression of Col1A1 and the levels of LAP-TGFβ-1 in nFB, suggesting that downregulation of FN1 by BioD is one of the primary underlying mechanisms of attenuated pro-fibrotic responses in keloid fibroblasts. This study reports that a decellularized ECM scaffold may significantly attenuate pro-fibrotic responses in both normal and keloid fibroblasts via TSP1 and FN1-dependent mechanisms.

## 1. Introduction

Biologic scaffolds derived from decellularized mammalian tissues consist of the extracellular matrix (ECM). They are instrumental in repairing a wide range of damaged or diseased tissues, including cardiac, esophageal, dermal, and musculotendinous tissues [1]. The production of ECM bio-scaffolds for tissue repair and regeneration entails the decellularization of the tissue or organ from which the ECM is to be extracted [2]. This decellularization process yields an acellular ECM scaffold devoid of xenogeneic and allogenic antigens [3]. These ECM-derived bio-scaffolds primarily contain matrix-associated structural and functional molecules that are mostly conserved across the species [4]. The small intestinal submucosa (SIS), a component of the small intestine, represents an ECM-based biomaterial that has found widespread use in tissue engineering and regenerative applications [5,6] due to non-immunogenicity [7]. It particularly exhibits advantages in promoting the healing of chronic wounds in humans [8,9]. Among other mechanisms of action of SIS, the immunomodulatory activity by an early transition of proinflammatory macrophage phenotype to anti-inflammatory macrophage phenotype, and the mobilization of progenitor cells are noteworthy [10]. Clinical studies performed using SIS compared to other single-component-based ECM biomaterials showed a clear advantage of SIS on wound-healing outcomes, esophageal, and dural repairs [11,12,13].

A dysfunctional ECM deposition and remodeling phase may result in an overabundance of ECM, causing fibrosis, scarring, and impaired tissue function [14]. Pathologic scarring is typically classified into two categories: (i) hypertrophic, which emerges within 4–8 weeks after wound closure and stabilizes over 6–8 months, and (ii) keloid, which extends beyond the original wound boundaries and does not diminish [15]. Keloids are more aggressive, fibroproliferative growths that arise from abnormal scarring processes, leading to an overproduction of fibrous tissue [16]. Keloid incidence varies by ethnicity, with higher rates in African Americans and Hispanics (4.5–6.2% to 16%) compared to Taiwanese, Chinese, and Caucasians (<1%) [17,18]. Various therapeutic approaches have been employed to treat pathologic scars, including steroid injections or surgical resection; however, frequent recurrences persist, necessitating a need for novel, effective treatment strategies for keloid management, specifically reduction in recurrences.

ECM-based materials have gained attention in scar management due to their potential to modulate the wound-healing process and thereby improve scar outcomes [19]. ECM biomaterials do more than just cover and protect wounds from external factors. They also function as a scaffold, promoting cell adhesion, proliferation, and differentiation, while guiding the development of healthy skin tissue [19]. In this study, our hypothesis focuses on the potential anti-fibrotic properties of SIS, with the goal of reducing pro-fibrotic responses in both human skin fibroblasts (nFB) and keloid fibroblasts (kFB). To test this hypothesis, the present study was conducted to assess how SIS influences the pro-fibrotic responses in nFB and kFB cells and to elucidate the underlying mechanisms governing this activity. nFBs used in this study were in a quiescent state and were not pre-activated with exogenous stimuli such as TGF-β before experimentation. Our rationale was guided by the primary objective of assessing the direct effects of BioD on quiescent fibroblasts, which better approximate the physiological state of dermal fibroblasts in intact tissue. To determine the effect of BioD in a fibrotic context, we also used kFB cells, fibroblasts derived from keloid skin. These cells are known to be in an activated state, exhibiting a pro-fibrotic phenotype characterized by excessive proliferation, migration, and extracellular matrix (ECM) production [20], providing a relevant model for evaluating the treatment’s anti-fibrotic responses.

## 2. Results

**Figure 1:**
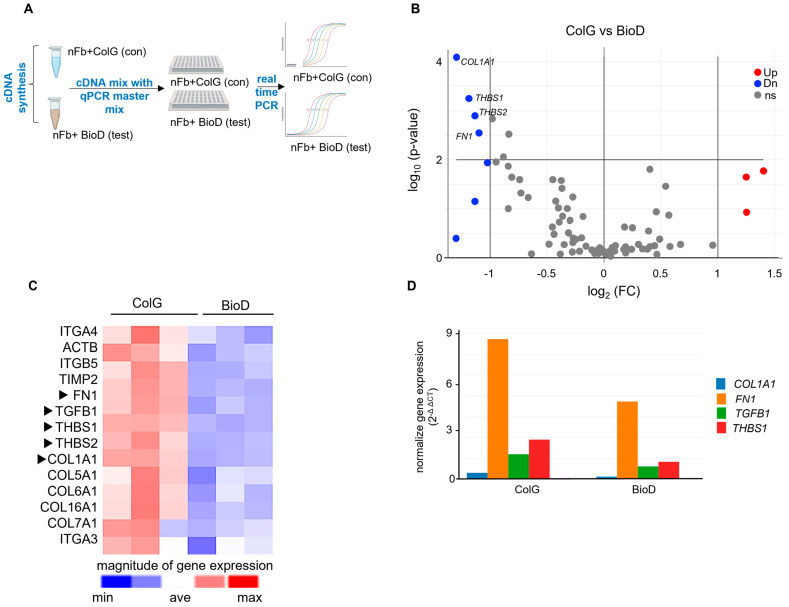
Effect of BioD on human ECM genes: RT2 Profiler PCR Array. Human normal fibroblasts (nFB) were either cultured on collagen gel (ColG) or on BioD for 48 h. Human extracellular matrix and adhesion molecules RT2 Profiler PCR Array was used to measure expression levels of 84 individual genes (Supplementary Table S1) for cell–cell adhesion and cell–matrix interactions. (**A**) Schema of the RT2 profiler experiment. (**B**) Volcano plot showing expression of differentially expressed up- (red) or down- (green) regulated genes (FC 2.0 and *p* < 0.01 were used as cutoff) in ColG vs. BioD groups. (**C**) Heatmap of all the significantly (*p* < 0.05) up- or downregulated genes in nFB cultured on ColG or BioD for 48 h. Arrow indicates the key pro-fibrotic genes shown in volcano plot. (**D**) Comparison of the expression level of each downregulated gene showed that *FN1* (orange) and *THBS1* (red) are predominant genes with high expression in nFB when compared with other significantly (*p* < 0.01) expressed pro-fibrotic pathway genes. FC, fold change.

BioD attenuates two key pro-fibrotic pathway genes in human normal and keloid fibroblasts. To perform a comprehensive quantitative screening of ECM-specific genes, we performed a human ECM and adhesion molecules RT2 Profiler PCR Array that simultaneously measures expression levels of eighty-four individual genes (Table S1) related to ECM, cell–cell adhesion, and cell–matrix interactions (Figure 1A). Data analysis was performed using RT2 Profiler PCR Array Data Analysis Web Portal (Qiagen, Germantown, MY, USA). A volcano plot was generated to visualize the differential gene expression of nFB cultured on BioD versus ColG for 48 h. The inclusion of the collagen gel (ColG) comparison group was based on its relevance as one of the most commonly used ECM-based biomaterials used for tissue engineering and injury repair [21]. BioD is primarily composed of type I collagen fibres [8]. Collagen-based matrices, including ColG, serve as widely accepted reference materials for evaluating biomaterial–cell interactions because they provide a well-characterized, fibroblast-responsive ECM environment. This comparison allowed us to determine whether the anti-fibrotic effects observed with BioD represent a material-specific property rather than a generic response to any collagen-containing substrate. The plot revealed that of the eighty-four transcripts examined (Table S1), seven were found to be downregulated with a fold change exceeding 1.5 and statistical significance at *p* < 0.01 (Figure 1B). In addition, a broader set of fourteen transcripts reached significance at the *p* < 0.05 level. A heat map has been presented to show this broader group of fourteen transcripts downregulated in nFB cultured on BioD as compared to those cultured on ColG (Figure 1C). This data suggests a specific cellular response of nFBs upon exposure to BioD.

Before advancing any further, we inquired whether the exposure to BioD had an impact on cell morphology or posed any toxicity to nFB. Cell morphology analysis was performed in culture. The CellTracker Orange cell tracker dye was used to visualize any changes in cell morphology or behavior in culture (Supplementary Figure S1A), while phalloidin stain was used for visualization of actin filaments and cytoskeletal changes (Supplementary Figure S1B). Phalloidin binds to F-actin filaments, which help visualize and examine the structural organization of the cytoskeleton, providing valuable insights into cell shape, size, and the arrangement of actin filaments. Finally, to determine any cytotoxic changes in BioD on nFB, we performed BV510 cell viability dye assay using flow cytometry. BV510, also known as Brilliant Violet 510, is a fluorochrome that emits blue-violet fluorescence when excited with a suitable laser [22] (Supplementary Figure S1C). None of these analyses showed any major cell behavior, structure, or loss in viability for nFB while cultured on BioD or ColG (Supplementary Figure S1).

In the RT2 Profiler experiment, a side-by-side bar graph plot of the absolute normalized expression values of the top five genes that were significantly different in BioD group as compared to ColG, which indicated an overall lower expression of *FN1* and *THBS1* genes in these cells (Figure 1D). Both genes play a significant role in fibrotic responses in keloid fibroblasts [23,24,25] and therefore, we focused our efforts on interrogating these two molecules in the following experiments.

**Figure 2:**
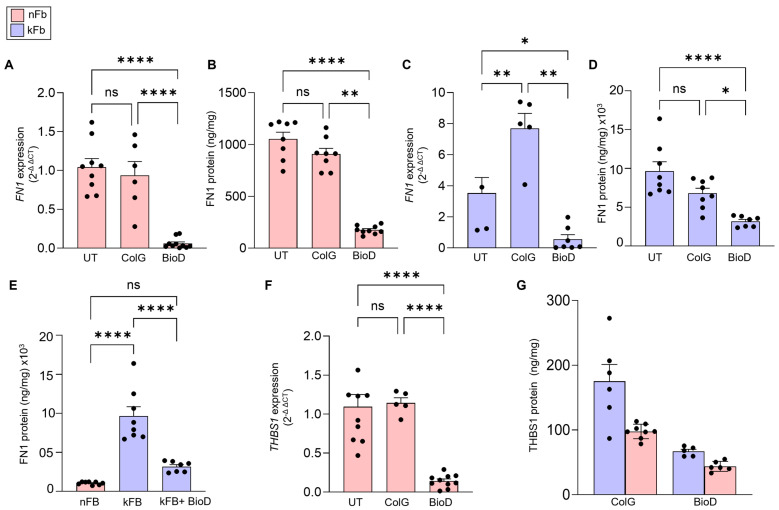
BioD attenuates fibronectin-1 and thrombospondin-1 expression mRNA and protein in normal and keloid fibroblasts. Human normal fibroblasts (nFB) or keloid fibroblasts (kFB) were cultured on untreated culture plates (UT), collagen-coated dishes (ColG), or on BioD for 48 h. (**A**) *FN1* mRNA; (**B**) FN1 protein in nFB; (**C**) *FN1* mRNA and (**D**) FN1 protein in kFB (**E**) comparison of FN1 protein in kFB vs. nFB and response to BioD; (**F**) *THBS1* mRNA and (**G**) THBS1 protein. The secreted ECM protein estimation was performed using ELISA, and mRNA expression was measured using real-time PCR. Data are mean ± SEM; * *p* < 0.05; ** *p* < 0.01, **** *p* < 0.0001; ns = not significant (*n* = 5–8 individual wells).

After selecting candidates from the RT2 Profiler array, we designed an experiment to validate the findings and assess the reproducibility of RT2Profiler data. Detailed independent validation experiments were performed on mRNA (real-time qPCR) and protein expression (ELISA) of fibronectin-1 and thrombospondin-1 in nFB cultured on BioD as compared to ColG (Figure 2A,B,F,G) showing that BioD significantly attenuated the expression of fibronectin-1 and thrombospondin-1 in these cells, confirming the screening data from RT2 profiler experiment. In addition to normal fibroblasts, we investigated the effect of BioD on the expression of fibronectin-1 and thrombospondin-1 in keloid-derived fibroblasts (kFB). Overproduction of ECM, including fibronectin-1 and thrombospondin-1, is a hallmark of keloid fibroblasts [23,26]. Exposure of kFB to BioD showed a marked reduction in fibronectin-1 mRNA and protein and thrombospondin-1 protein following 48 h of exposure (Figure 2C–E,G). Collectively, these data strongly indicate the potent efficacy of BioD in mitigating two pivotal molecules that regulate the pro-fibrotic pathway.

**Figure 3:**
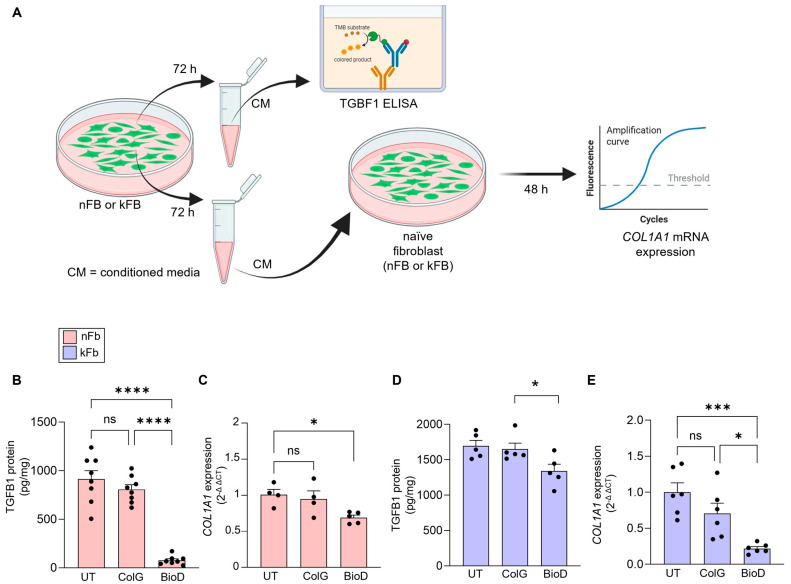
Conditioned media (CM) from fibroblast (nFB or kFB) cultured on BioD markedly inhibit the COL1A1 in naïve normal or keloid fibroblasts. nFB or kFB were cultured on untreated (UT), collagen-coated (ColG) dishes or with BioD. CM from these cells were collected after 72 h of culture followed by treatment to naïve normal or keloid fibroblasts. (**A**) schema of the experiment design. (**B**) TGFB1 protein levels in CM from nFB; (**C**) *COL1A1* in naïve nFB following treatment with CM from nFB; (**D**) TGFB1protein levels in CM from kFB; (**E**) *Col1A1* in naïve kFB following treatment with CM from kFB. The secreted ECM protein estimation was performed using ELISA, and mRNA expression was measured using real-time PCR. Data are mean ± SEM; * *p* < 0.05; *** *p* < 0.001 & **** *p* < 0.0001; ns = not significant (*n* = 4–8 individual wells).

Secreted factors released from human fibroblasts cultured on BioD attenuate TGFB1 and *COL1A1* in naïve fibroblasts. Transforming-growth factor-β (TGFB1 or TGFβ1) is a central regulator of ECM production and fibrosis in fibroblasts [27]. When activated, TGFB1 binds to its receptors on the cell surface, initiating a signalling cascade that includes Smad proteins [28]. When produced, TGFB1 is not in a biologically active form; it is released in a latent form consisting of TGFB1 and the non-covalently bound latency-associated peptide (LAP; derived from the N-terminal of the TGFB precursor), which must be released for activation [28]. THBS1 is one of the few known molecules that is capable of binding to latent TGFB, resulting in activation through the initiation of a conformational shift [29]. Based on this, we asked if BioD-mediated inhibition of THBS1 can affect the biological activity of TGFB1 released by nFB or kFB. Conditioned media (CM) from nFB or kFB was prepared by culturing these cells in the presence of BioD or ColG for 72 h (Figure 3A). Total TGFB1 levels in CM were assessed using ELISA. The CM derived from both nFB and kFB grown on BioD exhibited significantly reduced total TGFB1 levels (Figure 3B,D). To evaluate biological activity, we cultured naïve nFB and kFB with CM obtained from nFB and kFB cultured on BioD/ColG, respectively. We assessed *COL1A1* gene expression, a downstream product of the TGFB1 → CTGF (connective tissue growth factor—CTGF) signalling pathway [30]. The data not only illustrated decreased TGFB1 levels in the CM but also indicated diminished biological activity, suggesting a potential role of THBS1 in TGFB1 → *COL1A1* → fibrosis (Figure 3C,E). 

**Figure 4:**
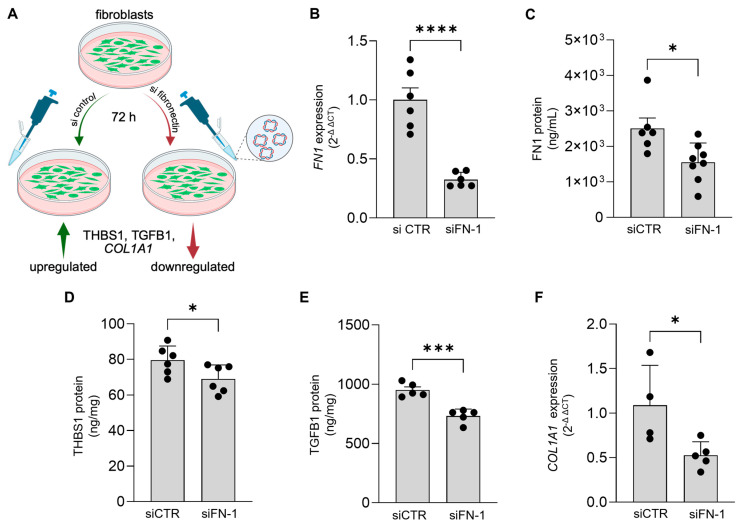
Knockdown of FN1 in nFB significantly attenuates THBS1 and pro-fibrotic pathway molecules, COL1A1 and TGFB1. Human normal fibroblasts (nFB) were subjected to siRNA-mediated knockdown of FN1, followed by assessment of THBS1 and pro-fibrotic pathway genes. (**A**) Schema showing knockdown of FN1 by siRNA, CTR denotes control siRNA; (**B**) knockdown of *FN1* by siRNA significantly reduced *FN1* mRNA expression in nFB, as well as (**C**) FN1 protein. (**D**) THBS1 protein (**E**) TGFB1 protein (**F**) *COL1A1* mRNA expression in nFB following FN1 knockdown. The secreted ECM protein estimation was performed using ELISA, and mRNA expression was measured using real-time PCR. Data are mean ± SEM; * *p* < 0.05; *** *p* < 0.001 & **** *p* < 0.0001 *(n* = 4–6 individual wells).

Fibronectin-1 plays a central role in attenuating molecules of pro-fibrotic pathway. Fibronectin-1 is a glycoprotein that plays a crucial role in the ECM and tissue remodelling [31]. In fibrotic conditions, such as keloids, fibronectin-1 is often overexpressed and contributes to the excessive deposition of ECM components, primarily collagen [23]. We investigated the role of *FN1* on the expression of THBS1 and pro-fibrotic factors TGFB1 and *COL1A1*, responsive to BioD (Figure 4). Knockdown of *FN1* using siRNA-based approach resulted in significant attenuation of fibronectin in nFB (Figure 4B,C). Such knockdown also resulted in reduction in the expression of THBS1, TGFB1 and *COL1A1* (Figure 4D–F), suggesting a role of *FN1* in the regulation of key molecules of the fibroblast fibrosis response. BioD is known to contain native fibronectin-1 [32]. To determine if the lowering of FN1 by BioD is interfered by the endogenous FN1 in BioD, we prepared CM from cell-free BioD and ColG by treating the biomaterials with cell culture media, followed by determining the levels of FN1 in CM derived from cell-free BioD (Supplementary Figure S2A). The measured amounts were 556.5 pg/mL and 248.5 pg/mL, respectively, in BioD or ColG cell-free CM. Recombinant FN1 amount (308 pm/mL) that was excess in the BioD group was added to ColG in CM, followed by treatment of naïve nFB, followed by determination of FN1 levels (Supplementary Figure S2C) which showed no changes in the levels of FN1. However, THBS1 level was significantly downregulated (Supplementary Figure S2B), suggesting that the endogenous levels of FN1 do not interfere with the effect observed in nFB following exposure to BioD. Secreted ECM protein levels were measured by Enzyme-Linked Immunosorbent Assay (ELISA), and mRNA expression was measured by real-time PCR.

## 3. Discussion

Keloidal scarring is a challenging skin disease due to its persistent growth and aggressive nature. Recently, there has been a growing interest in the use of biomaterials and scaffolds mimicking ECM for scar and keloid management [19]. The proposed mechanisms by which these materials improve scars are primarily by facilitating angiogenesis, reduction in inflammation response, and regulating ECM deposition [19]. Nevertheless, the precise molecular mechanisms governing their effects, particularly regarding the regulation of ECM deposition by fibroblasts, remain unclear. This current study breaks new ground by identifying key molecules within the fibrotic pathway that are significantly suppressed by BioD in fibroblasts. The research sheds light on the central roles played by fibronectin-1 and thrombospondin-1 in orchestrating the anti-fibrotic response induced by exposure to BioD in both normal and keloid fibroblasts.

Pathological scarring, seen in hypertrophic or keloid scars, involves fibroblasts excessively producing ECM components, particularly type I collagen. Multi-omics investigations, including proteomics, have identified abundant levels of collagens (I and III), FN1, THBS1, and TGFB1 in keloids compared to normal skin [33]. Interestingly, our current study has revealed the suppression of these molecules by BioD (Figure 1). Of these molecules, THBS1 and FN1 were focus of the current study because of their regulatory role in fibrosis, and their expression levels were higher in untreated fibroblasts when compared to other molecules that were targeted by BioD.

Thrombospondin-1 expression by keloid fibroblasts is quantitatively more than the normal fibroblasts [24]. The mechanism by which THBS1 regulates fibrosis and tissue repair includes stimulating intermediate collagen production and cell adhesion through calreticulin (CRT)–LDL-receptor-related protein (LRP1) as well as regulating latent transforming-growth factor β activation [34]. A central role of thrombospondin-1 in TGFb1/Smad3 signalling pathway and subsequent fibrosis has been established [35,36].The use of approaches with thrombospondin-1 inhibitory activity has been proposed as a novel therapeutic modality against fibrosis [36]. Current work identifies a decellularized ECM biomaterial as an effective thrombospondin-1 inhibitor that may be used to prevent a pro-fibrotic response in injured tissues.

Keloid fibroblasts are also reported to exhibit excessive fibronectin-1 production via stimulation of the transcription process [23,37]. TGFB1 is recognized as a central mediator responsible for initiating and sustaining the fibrotic response in hypertrophic scars [38] and keloids [39,40]. *COL1A1* is transcriptionally regulated by *TGFB1* [41,42]. Both fibronectin-1 and thrombospondin-1 are acknowledged for their involvement in regulating TGFB1 expression and activation levels [29,43]. Conversely, there is a reciprocal relationship, with TGFB1 also influencing *FN1* and *THBS1* gene expressions [44]. No significant differences were noted between the untreated and ColG-treated fibroblasts. ECM, including collagen membranes, is known to naturally contain bound growth factors, such as TGF-β [45]. However, reported levels of TGF-β released from collagen membranes vary widely, ranging from no detection to picogram to nanogram quantities. Moreover, several studies indicate that collagenase-mediated activation is required to facilitate measurable release [45,46]. It is therefore plausible that, in our system, TGF-β levels were either below the limit of detection or insufficient due to the lack of an active release mechanism. Consistent with these findings, we also observed negligible TGF-β release from BioD, despite prior reports of TGF-β in this material [47].

In keloids, the primary genetically distinct collagen types include I, III, and V [33]. Our gene expression profiling data have revealed a significant suppression of multiple collagen transcripts, including *COL1A1*, *COL4A2*, *COL5A1*, *COL6A1*, *COL6A2*, and *COL7A1*. Among these transcripts, the most significant reduction was observed in the expression of *COL1A1* transcript.

In keloids, knockdown of fibronectin-1 has been shown to inhibit TGFB1-mediated cell proliferation and collagen deposition via AKT/ERK signalling pathway [48]. The *FN1* knockdown experiments in this study align with the earlier observations, as the attenuation of *FN1* via siRNA transfection resulted in a significant decrease in the levels of TGFB1 protein (Figure 4E) and *COL1A1* mRNA expression (Figure 4F). We also noted that knockdown of *FN1* significantly reduced THBS1 protein levels (Figure 4D). These observations underscore a central role of fibronectin-1 in the BioD-mediated anti-fibrotic response observed in fibroblasts. In this study, knockdown of FN1 was performed in nFBs, while the findings offer valuable insights into the regulatory role of FN1 on THBS1 protein in nFB, their extrapolation to kFB should be approached with caution, as the regulatory mechanisms in pathogenic cells may differ markedly. Therefore, further studies using disease-relevant cell models are necessary to determine whether the observed regulatory effects are preserved or modified in the pathological state.

The influence of exposure to BioD on various highly specific subsets of genes related to the ECM and associated molecules indicates that the underlying molecular mechanisms of this effect are indeed distinctive and unparalleled. Upon reviewing the existing literature, several potential mechanisms driving the anti-fibrotic effects of BioD emerge as plausible. Firstly, the mechanical attributes of the ECM may play a pivotal role by recreating the fibroblasts’ native environment and maintaining them in a non-activated state [49]. Secondly, it is likely that the presence of growth factors, such as fibroblast growth factor (FGF2) and activities related to TGFB, which are associated with SIS, contribute to these effects [50]. Additionally, biologically active TGFB1 has been reported to be associated with sterilized SIS [51]. Of the two likely mechanisms mentioned above, the mechanical attributes are unlikely to play a substantial role in the current study, as our experimental data indicate that bioscaffolds such as Integra or collagen gel were unable to replicate the inhibitory effects on thrombospondin-1 or fibronectin-1 that were observed with SIS. Furthermore, the pro-fibrotic effects of two growth factors FGF2 and TGFB1 are well-known and therefore, it is unlikely that these growth factors in BioD influenced the observed anti-fibrotic effects of BioD. CM derived from cell-free BioD demonstrated the ability to induce an anti-fibrotic response at least partially in both naïve nFB and kFB, suggesting that certain factors present in the CM may contribute to the effects of BioD. More recently, matrix-bound nanovesicles (MBVs) have been acknowledged as an integral and active component of ECM bioscaffolds [52]. It is conceivable that the MBVs released by BioD contain certain bioactive molecules that, at least partially, contribute to the anti-fibrotic effect on nFB and kFB.

Conclusions. In summary, this study has presented significant evidence of a dampened fibroblast response in both normal and keloid fibroblasts when exposed to BioD. Furthermore, this study has identified thrombospondin-1 or fibronectin-1 as pivotal hubs in the regulatory network through which BioD mediates its anti-fibrotic effects. Future animal and clinical studies are imperative to comprehensively elucidate the anti-fibrotic potential of BioD in the management of keloids. Additionally, this study has shed light on potential novel mechanisms of action of BioD, including the regulation of anti-fibrotic responses through secretome (likely MVB) from cell-free BioD, which warrants further investigation.

Limitations and future scope of study. This study is limited by its reliance on cultured cell models. While these systems allow controlled, mechanistic investigations, they may not fully reflect in vivo biology. The simplified in vitro environment lacks the complex cellular, matrix, and immunological interactions present in living tissues. Furthermore, the studies used type 1 collagen-coated surfaces (ColG) as a control comparator for the BioD-mediated anti-fibrosis responses. Although ColG provided a practical and biologically relevant benchmark, a “perfect” control for biomaterials such as BioD is inherently difficult to define. Available scaffolds vary widely in biochemical composition, degree and type of cross-linking, and processing or sterilization methods—factors that can independently influence cellular responses—making it challenging to identify a single ideal reference material. The current studies evaluated the effects of FN1 knockdown on fibrosis response before normal fibroblasts had fully acquired a keloid-like phenotype. Direct targeting of FN1 in pro-fibrotic keloid fibroblasts (kFB) may provide additional insight into the central role of FN1 in mediating BioD’s anti-fibrosis response. Taken together, the findings from this study warrant further investigation into the anti-fibrotic effects of BioD and the mechanistic underpinnings of its activity in relevant in vivo models of hypertrophic scarring and keloid formation.

## 4. Materials and Methods

### 4.1. Cell Culture

An immortalized human fibroblast cell line or nFB (BJ-5ta-CRL-4001, ATCC, Manassas, VA, USA) was grown under standard culture conditions (at 37 °C in a humidified atmosphere consisting of 95% air and 5% CO_2_) in a 4:1 mixture of Dulbecco’s Modified Eagle’s Medium (DMEM, Cat# 30-2002, ATCC, VA, USA) and Medium 199 (Cat#1 11150067, ThermoFisher Scientific, Waltham, MA, USA) supplemented with 0.1 mg/mL penicillin–streptomycin (Cat#15140122, ThermoFisher Scientific, Waltham, MA, USA) and 10% fetal bovine serum (Cat# 16000044, ThermoFisher Scientific, MA, USA). These nFB cells are immortalized via the introduction of telomerase reverse transcriptase, hTERT [53]. Throughout the manuscript, nFB refers to immortalized human normal foreskin fibroblasts.

Human keloid fibroblast cell line or, kFB (KEL FIB-CRL-1762, ATCC, VA, USA) isolated from the connective tissue of a 35-year-old black female’s skin with keloid, was grown in similar condition as the BJ-5ta cells, in Dulbecco’s Modified Eagle’s Medium (Cat# 30-2002, ATCC, VA, USA), supplemented with 10% FBS, 100 IU/mL antimicrobial-antimycotic, (Cat#15240062, ThermoFisher Scientific, Waltham, MA, USA) [54].

### 4.2. Cell Culture on Biodesign (BioD)

Biodesign (BioD), is an ECM-based biomaterial-derived from naturally occurring extracellular matrices of porcine small intestinal submucosa (SIS, Cook Biotech Inc., Indianapolis, IN, USA) [55,56]. For treatment, human normal or keloid fibroblasts were cultured on BioD for the specified time, and the control groups consisted of cells cultured on either collagen gel, referred to as ColG (Cat# A1048301, ThermoFisher Scientific, MA, USA), or Integra Matrix cross-linked bovine tendon collagen and glycosaminoglycan wound dressings (Integra LifeSciences, Princeton, NJ, USA). We cultured the cells for 48 h for the RT2Profiler array and its validation as per the prior studies, which have shown that fibroblast activation, ECM remodelling signals, and early pro-fibrotic gene/protein expression changes typically emerge within 24–48 h following exposure to extracellular cues or biomaterials [57,58]. By 48 h, these responses are sufficiently established to enable reliable detection, while still reflecting early-stage signalling rather than secondary or downstream adaptive changes. Additionally, preliminary optimization experiments in our laboratory indicated that 48 h provided consistent cell viability (shown in Figure S1C) and robust RNA yield across all experimental groups, ensuring comparable conditions for differential gene expression analysis.

### 4.3. Cell Viability

The viability of nFB cells was determined by staining the cells with BD Horizon Fixable Viability Stain-510 in serum-free buffer, 1000× dilution (Cat# 564406, BD Biosciences, Franklin Lakes, NJ, USA) and flow cytometry analysis. Cells were gated using forward and side scatter characteristics with at least 10,000 gated events recorded using violet laser-equipped flow cytometer BD LSRFortessa (BD Biosciences, NJ, USA) and analyzed using FlowJo v10 software.

### 4.4. Cell Behavior, Morphology and Viability

nFB cells were labeled with 5 µM CellTracker Orange CMTMR Dye (Cat# C2927, ThermoFisher Scientific, Waltham, MA, USA) for 45 min. Nuclei were counter-stained with Hoechst (2 µg/mL) for 15 min. The Cell Tracker-labeled nFB cells were seeded onto BioD or control matrices as indicated before. Images were obtained using 63× oil immersion objective and a confocal microscope (LSM880, Carl Zeiss, Oberkochen, Germany) to observe cell behavior in vivo. To visualize cell morphology and cytoskeleton, the cells were fixed with 4% paraformaldehyde and permeabilized using 0.1% Triton X-100. Phalloidin conjugate (Cat# A12380, ThermoFisher Scientific, Waltham, MA, USA) working solution (1×) was added to the cells, incubated for 90 min at room temperature, and counterstained with Hoechst (2 µg/mL for 15 min). Images were obtained using a 63× oil immersion objective and a confocal microscope (LSM880, Carl Zeiss). Phalloidin binds F-actin with high selectivity, while Alexa Fluor 568 provides red fluorescence [59,60].

### 4.5. RNA Isolation, Reverse Transcription, and Quantitative RT-PCR (qRT-PCR) and RT2 Profiler PCR Array

Total RNA was extracted using miRVana RNA isolation kit (Cat# AM1560, Thermo Scientific, Waltham, MA, USA) as per the manufacturer’s protocol as previously described [61]. RNA concentration and purity were assessed on NanoDrop 1000 (ThermoFisher Scientific, Waltham, MA, USA).

The Quantabio qScript cDNA Synthesis Kit (Cat# 95047-100, Quanta, Houston, TX, USA) was used to generate first-strand cDNA. Gene expression was measured by real-time qPCR assay using DNA intercalation dye PowerupSYBR Green (Applied Biosystems, Waltham, MA, USA) [59]. Gene-specific primers were used for the assay. For the RT2 profiler, RNA was reverse-transcribed to cDNA using RT2 First Strand Kit (Cat# 330404, Qiagen, Valencia, CA, USA). The human extracellular matrix (hECM) and Adhesion Molecules RT2 Profiler PCR Array (PAHS-013Z, Cat# 330231; Qiagen, Valencia, CA, USA) was used to determine expression levels of 84 individual genes crucial for cell–cell and cell–matrix interactions. Fluorescent signal was captured using ABI 7900 Real-Time PCR System (Applied Biosystems, Life Technologies, Carlsbad, CA, USA) [62].

### 4.6. Enzyme-Linked Immunoassay (ELISA)

Levels of thrombospondin-1 (THBS1; Cat# DY3074; R&D, Minneapolis, MN, USA), transforming-growth factor beta-1 (TGFB1; DY24605, R&D, MN, USA) and fibronectin-1 (FN1; Cat# ab219046; Abcam, Waltham, MA, USA) secreted by human fibroblast cells were measured using commercially available ELISA kits as per manufacturer’s protocols. For the assay, the cells were seeded on 12-well plates, and the media were collected after a specified time to perform ELISA. Briefly, 96-well plates were coated with capture antibodies overnight at room temperature and blocked with 2% BSA in phosphate-buffered saline (PBS) followed by extensive wash steps. The protein standards and samples were applied and incubated for 2 h. Next, plates were incubated with biotin-conjugated detection antibodies for 2 h and streptavidin-HRP for 30 min at room temperature. The signal was developed with TMB substrate (Thermo Scientific, MA, USA), and 450 nm absorbance was measured on a BioTEK HT plate reader (Tecan, NC, USA). Concentrations were calculated according to the respective standard curves.

### 4.7. siRNA Transfection

DharmaFECT1 transfection reagent (Dharmacon RNA technologies, Lafayette, CO, USA) was used to transfect cells with ON-TARGETplus siRNA FN1 Smartpool (Cat# L-009853-00-0005; Dharmacon RNA technologies, USA) for 72 h as per the manufacturer’s instructions, as previously described [63]. ON-TARGETplus Non-targeting Control Pool (Cat# D-001810-10-20; Dharmacon. RNA Technologies, USA) was used for control transfections. In brief, DharmaFECT 1 was used to transfect cells with a 100 nM siRNA pool of *FN1* for 72 h. For control, siControl non-targeting siRNA pool (mixture of 4 siRNA, designed to have ≥4 mismatches with the gene) were used. Using this approach, the transfection efficiency was ~70% [64].

### 4.8. Statistical Analysis

Statistical analysis was performed using GraphPad Prism 8 software. Data distribution was calculated using the Shapiro–Wilk test. Normally distributed data were presented as mean ± standard deviation and analyzed by unpaired parametric *t*-test. For comparisons of more than two groups, an ANOVA test with multiple comparisons (normally distributed data) was performed. Data are reported as mean ± SEM as indicated in the respective figure legends. The *p*-value less than 0.05 was considered statistically significant.

## Data Availability

The data that support the findings of this study are available on request from the corresponding author, Sashwati Roy.

## Supplementary Materials

The following supporting information can be downloaded at https://www.mdpi.com/article/10.3390/ijms262411764/s1.

## Abbreviations

The following abbreviations are used in this manuscript:

BioD	Biodesign
CM	conditioned media
ColG	collagen gel
CTR	control
ECM	extracellular matrix
FN	fibronectin
LAP	latency-associated peptide
MVB	multivesicular bodies
nFB	human keloid fibroblast
nFB	normal human fibroblast
SIS	small intestinal submucosa
TGF	transforming-growth factor
THBS	thrombospondin

## References

[1] Badylak: S.F., Brown B.N., Gilbert T.W., Daly K.A., Huber A., Turner N.J. (2011). Biologic scaffolds for constructive tissue remodeling. Biomaterials.

[2] Crapo P.M., Gilbert T.W., Badylak S.F. (2011). An overview of tissue and whole organ decellularization processes. Biomaterials.

[3] Keane T.J., Londono R., Turner N.J., Badylak S.F. (2012). Consequences of ineffective decellularization of biologic scaffolds on the host response. Biomaterials.

[4] Brown N.H. (2011). Extracellular matrix in development: Insights from mechanisms conserved between invertebrates and vertebrates. Cold Spring Harb. Perspect. Biol..

[5] Zhang F., Zhang J., Lin S., Oswald T., Sones W., Cai Z., Dorsett-Martin W., Lineaweaver W.C. (2003). Small intestinal submucosa in abdominal wall repair after TRAM flap harvesting in a rat model. Plast. Reconstr. Surg..

[6] Campodonico F., Benelli R., Michelazzi A., Ognio E., Toncini C., Maffezzini M. (2004). Bladder cell culture on small intestinal submucosa as bioscaffold: Experimental study on engineered urothelial grafts. Eur. Urol..

[7] Badylak S.F. (2004). Xenogeneic extracellular matrix as a scaffold for tissue reconstruction. Transpl. Immunol..

[8] Shi L., Ronfard V. (2013). Biochemical and biomechanical characterization of porcine small intestinal submucosa (SIS): A mini review. Int. J. Burn. Trauma.

[9] Kim H.S., Hwang H.J., Kim H.J., Choi Y., Lee D., Jung H.H., Do S.H. (2022). Effect of Decellularized Extracellular Matrix Bioscaffolds Derived from Fibroblasts on Skin Wound Healing and Remodeling. Front. Bioeng. Biotechnol..

[10] Dziki J.L., Sicari B.M., Wolf M.T., Cramer M.C., Badylak S.F. (2016). Immunomodulation and Mobilization of Progenitor Cells by Extracellular Matrix Bioscaffolds for Volumetric Muscle Loss Treatment. Tissue Eng. Part A.

[11] Badylak S.F., Hoppo T., Nieponice A., Gilbert T.W., Davison J.M., Jobe B.A. (2011). Esophageal preservation in five male patients after endoscopic inner-layer circumferential resection in the setting of superficial cancer: A regenerative medicine approach with a biologic scaffold. Tissue Eng. Part A.

[12] Bejjani G.K., Zabramski J. (2007). Safety and efficacy of the porcine small intestinal submucosa dural substitute: Results of a prospective multicenter study and literature review. J. Neurosurg..

[13] Xiao H., Chen X., Liu X., Wen G., Yu Y. (2023). Recent advances in decellularized biomaterials for wound healing. Mater. Today Bio.

[14] Jun J.-I., Lau L.F. (2010). Cellular senescence controls fibrosis in wound healing. Aging (Albany NY).

[15] Limandjaja G.C., Niessen F.B., Scheper R.J., Gibbs S. (2021). Hypertrophic scars and keloids: Overview of the evidence and practical guide for differentiating between these abnormal scars. Exp. Dermatol..

[16] Viera M.H., Vivas A.C., Berman B. (2012). Update on Keloid Management: Clinical and Basic Science Advances. Adv. Wound Care (New Rochelle).

[17] Rockwell W.B., Cohen I.K., Ehrlich H.P. (1989). Keloids and hypertrophic scars: A comprehensive review. Plast. Reconstr. Surg..

[18] Sun L.-M., Wang K.-H., Lee Y.-C.G. (2014). Keloid incidence in Asian people and its comorbidity with other fibrosis-related diseases: A nationwide population-based study. Arch. Dermatol. Res..

[19] Zhou S., Wang Q., Huang A., Fan H., Yan S., Zhang Q. (2021). Advances in Skin Wound and Scar Repair by Polymer Scaffolds. Molecules.

[20] Bettinger D.A., Yager D.R., Diegelmann R.F., Cohen I.K. (1996). The effect of TGF-beta on keloid fibroblast proliferation and collagen synthesis. Plast. Reconstr. Surg..

[21] Rezvani Ghomi E., Nourbakhsh N., Akbari Kenari M., Zare M., Ramakrishna S. (2021). Collagen-based biomaterials for biomedical applications. J. Biomed. Mater. Res. Part B Appl. Biomater..

[22] Zhang X., Lu X., Moog C., Yuan L., Liu Z., Li Z., Xia W., Zhou Y., Wu H., Zhang T. (2018). KIR3DL1-Negative CD8 T Cells and KIR3DL1-Negative Natural Killer Cells Contribute to the Advantageous Control of Early Human Immunodeficiency Virus Type 1 Infection in HLA-B Bw4 Homozygous Individuals. Front. Immunol..

[23] Babu M., Diegelmann R., Oliver N. (1989). Fibronectin is overproduced by keloid fibroblasts during abnormal wound healing. Mol. Cell. Biol..

[24] Centeno R.F., Albo D., Rothman V.L., Granick M.S., Tuszynski G.P. (2002). Thrombospondin-1 May Modulate Keloid Formation Through Up-Regulation of the Matrix-Associated Plasminogen/Plasmin System. Plast. Reconstr. Surg..

[25] Feng Q.L., Gu J.J., Chen J.Y., Zheng W.Y., Pan H.H., Xu X.Y., Deng C.C., Yang B. (2022). TSP1 promotes fibroblast proliferation and extracellular matrix deposition via the IL6/JAK2/STAT3 signalling pathway in keloids. Exp. Dermatol..

[26] Jiang D., Guo B., Lin F., Hui Q., Tao K. (2020). Effect of THBS1 on the Biological Function of Hypertrophic Scar Fibroblasts. BioMed Res. Int..

[27] Moses H.L., Roberts A.B., Derynck R. (2016). The Discovery and Early Days of TGF-β: A Historical Perspective. Cold Spring Harb. Perspect. Biol..

[28] Jia S., Meng A. (2021). TGFβ family signaling and development. Development.

[29] Ribeiro S.M., Poczatek M., Schultz-Cherry S., Villain M., Murphy-Ullrich J.E. (1999). The activation sequence of thrombospondin-1 interacts with the latency-associated peptide to regulate activation of latent transforming growth factor-beta. J. Biol. Chem..

[30] Hillege M.M.G., Galli Caro R.A., Offringa C., de Wit G.M.J., Jaspers R.T., Hoogaars W.M.H. (2020). TGF-β Regulates Collagen Type I Expression in Myoblasts and Myotubes via Transient *Ctgf* and *Fgf-2* Expression. Cells.

[31] Schwarzbauer J.E., DeSimone D.W. (2011). Fibronectins, their fibrillogenesis, and in vivo functions. Cold Spring Harb. Perspect. Biol..

[32] Hodde J., Janis A., Ernst D., Zopf D., Sherman D., Johnson C. (2007). Effects of sterilization on an extracellular matrix scaffold: Part I. Composition and matrix architecture. J. Mater. Sci. Mater. Med..

[33] Bakhtyar N., Amini-Nik S., Jeschke M.G. (2022). OMICS Approaches Evaluating Keloid and Hypertrophic Scars. Int. J. Inflamm..

[34] Sakai K., Sumi Y., Muramatsu H., Hata K., Muramatsu T., Ueda M. (2003). Thrombospondin-1 promotes fibroblast-mediated collagen gel contraction caused by activation of latent transforming growth factor beta-1. J. Dermatol. Sci..

[35] Atanasova V.S., Russell R.J., Webster T.G., Cao Q., Agarwal P., Lim Y.Z., Krishnan S., Fuentes I., Guttmann-Gruber C., McGrath J.A. (2019). Thrombospondin-1 Is a Major Activator of TGF-beta Signaling in Recessive Dystrophic Epidermolysis Bullosa Fibroblasts. J. Investig. Dermatol..

[36] Murphy-Ullrich J.E. (2019). Thrombospondin 1 and Its Diverse Roles as a Regulator of Extracellular Matrix in Fibrotic Disease. J. Histochem. Cytochem..

[37] Oliver N., Babu M., Diegelmann R. (1992). Fibronectin gene transcription is enhanced in abnormal wound healing. J. Investig. Dermatol..

[38] Zhang K., Garner W., Cohen L., Rodriguez J., Phan S. (1995). Increased types I and III collagen and transforming growth factor-β1 mRNA and protein in hypertrophic burn scar. J. Investig. Dermatol..

[39] Elsaie M.L. (2021). Update on management of keloid and hypertrophic scars: A systemic review. J. Cosmet. Dermatol..

[40] So K., McGrouther D.A., Bush J.A., Durani P., Taylor L., Skotny G., Mason T., Metcalfe A., O’Kane S., Ferguson M.W.J. (2011). Avotermin for Scar Improvement following Scar Revision Surgery: A Randomized, Double-Blind, Within-Patient, Placebo-Controlled, Phase II Clinical Trial. Plast. Reconstr. Surg..

[41] Pan X., Chen Z., Huang R., Yao Y., Ma G. (2013). Transforming growth factor beta1 induces the expression of collagen type I by DNA methylation in cardiac fibroblasts. PLoS ONE.

[42] Jimenez S.A., Varga J., Olsen A., Li L., Diaz A., Herhal J., Koch J. (1994). Functional analysis of human alpha 1(I) procollagen gene promoter. Differential activity in collagen-producing and -nonproducing cells and response to transforming growth factor beta 1. J. Biol. Chem..

[43] Zhang H., Chen X., Xue P., Ma X., Li J., Zhang J. (2021). FN1 promotes chondrocyte differentiation and collagen production via TGF-β/PI3K/Akt pathway in mice with femoral fracture. Gene.

[44] Ranganathan P., Agrawal A., Bhushan R., Chavalmane A.K., Kalathur R.K., Takahashi T., Kondaiah P. (2007). Expression profiling of genes regulated by TGF-beta: Differential regulation in normal and tumour cells. BMC Genom..

[45] Panahipour L., Kargarpour Z., Luza B., Lee J.S., Gruber R. (2020). TGF-beta Activity Related to the Use of Collagen Membranes: In Vitro Bioassays. Int. J. Mol. Sci..

[46] Di Summa F., Kargarpour Z., Nasirzade J., Stahli A., Mitulovic G., Panic-Jankovic T., Koller V., Kaltenbach C., Muller H., Panahipour L. (2020). TGFbeta activity released from platelet-rich fibrin adsorbs to titanium surface and collagen membranes. Sci. Rep..

[47] Orabi H., Safwat A.S., Shahat A., Hammouda H.M. (2013). The use of small intestinal submucosa graft for hypospadias repair: Pilot study. Arab J. Urol..

[48] Cui J., Li Z., Jin C., Jin Z. (2020). Knockdown of fibronectin extra domain B suppresses TGF-β1-mediated cell proliferation and collagen deposition in keloid fibroblasts via AKT/ERK signaling pathway. Biochem. Biophys. Res. Commun..

[49] Halliday N.L., Tomasek J.J. (1995). Mechanical properties of the extracellular matrix influence fibronectin fibril assembly in vitro. Exp. Cell Res..

[50] Voytik-Harbin S.L., Brightman A.O., Kraine M.R., Waisner B., Badylak S.F. (1997). Identification of extractable growth factors from small intestinal submucosa. J. Cell. Biochem..

[51] McDevitt C.A., Wildey G.M., Cutrone R.M. (2003). Transforming growth factor-beta1 in a sterilized tissue derived from the pig small intestine submucosa. J. Biomed. Mater. Res. A.

[52] Huleihel L., Hussey G.S., Naranjo J.D., Zhang L., Dziki J.L., Turner N.J., Stolz D.B., Badylak S.F. (2016). Matrix-bound nanovesicles within ECM bioscaffolds. Sci. Adv..

[53] Janeckova L., Pospichalova V., Fafilek B., Vojtechova M., Tureckova J., Dobes J., Dubuissez M., Leprince D., Baloghova N., Horazna M. (2015). HIC1 Tumor Suppressor Loss Potentiates TLR2/NF-κB Signaling and Promotes Tissue Damage-Associated Tumorigenesis. Mol. Cancer Res..

[54] Roy S., Santra S., Das A., Dixith S., Sinha M., Ghatak S., Ghosh N., Banerjee P., Khanna S., Mathew-Steiner S. (2020). Staphylococcus aureus Biofilm Infection Compromises Wound Healing by Causing Deficiencies in Granulation Tissue Collagen. Ann. Surg..

[55] Fujii M., Tanaka R. (2022). Porcine Small Intestinal Submucosa Alters the Biochemical Properties of Wound Healing: A Narrative Review. Biomedicines.

[56] Aachoui Y., Ghosh S.K. (2011). Extracellular matrix from porcine small intestinal submucosa (SIS) as immune adjuvants. PLoS ONE.

[57] Karhu S.T., Ruskoaho H., Talman V. (2021). Distinct Regulation of Cardiac Fibroblast Proliferation and Transdifferentiation by Classical and Novel Protein Kinase C Isoforms: Possible Implications for New Antifibrotic Therapies. Mol. Pharmacol..

[58] Fang F., Ooka K., Bhattacharyya S., Wei J., Wu M., Du P., Lin S., Del Galdo F., Feghali-Bostwick C.A., Varga J. (2011). The early growth response gene Egr2 (Alias Krox20) is a novel transcriptional target of transforming growth factor-beta that is up-regulated in systemic sclerosis and mediates profibrotic responses. Am. J. Pathol..

[59] Roy S., Khanna S., Rink T., Radtke J., Williams W.T., Biswas S., Schnitt R., Strauch A.R., Sen C.K. (2007). P21waf1/cip1/sdi1 as a central regulator of inducible smooth muscle actin expression and differentiation of cardiac fibroblasts to myofibroblasts. Mol. Biol. Cell.

[60] Joshi B., Bastiani M., Strugnell S.S., Boscher C., Parton R.G., Nabi I.R. (2012). Phosphocaveolin-1 is a mechanotransducer that induces caveola biogenesis via Egr1 transcriptional regulation. J. Cell Biol..

[61] Ghosh N., Das A., Biswas N., Gnyawali S., Singh K., Gorain M., Polcyn C., Khanna S., Roy S., Sen C.K. (2020). Urolithin A augments angiogenic pathways in skeletal muscle by bolstering NAD^+^ and SIRT1. Sci. Rep..

[62] Sinha M., Sen C.K., Singh K., Das A., Ghatak S., Rhea B., Blackstone B., Powell H.M., Khanna S., Roy S. (2018). Direct conversion of injury-site myeloid cells to fibroblast-like cells of granulation tissue. Nat. Commun..

[63] Das A., Madeshiya A.K., Biswas N., Ghosh N., Gorain M., Rawat A., Mahajan S.P., Khanna S., Sen C.K., Roy S. (2022). Oncostatin M Improves Cutaneous Wound Re-Epithelialization and Is Deficient under Diabetic Conditions. J. Investig. Dermatol..

[64] Das A., Ganesh K., Khanna S., Sen C.K., Roy S. (2014). Engulfment of apoptotic cells by macrophages: A role of microRNA-21 in the resolution of wound inflammation. J. Immunol..

