# Supplementary material for: Anti-Fibrotic Properties of a Decellularized Extracellular Matrix Scaffold from Porcine Small Intestinal Submucosa in Normal Human and Keloid Fibroblasts
Source: Int J Mol Sci. 2025 Dec 5;26(24):11764. doi: 10.3390/ijms262411764 (PMC12732390; doi:10.3390/ijms262411764)
Supplement: Supplementary file 1 [file ijms-26-11764-s001.zip › ijms-3918056-supplementary.pdf]

Supplementary Figures

Figure S1

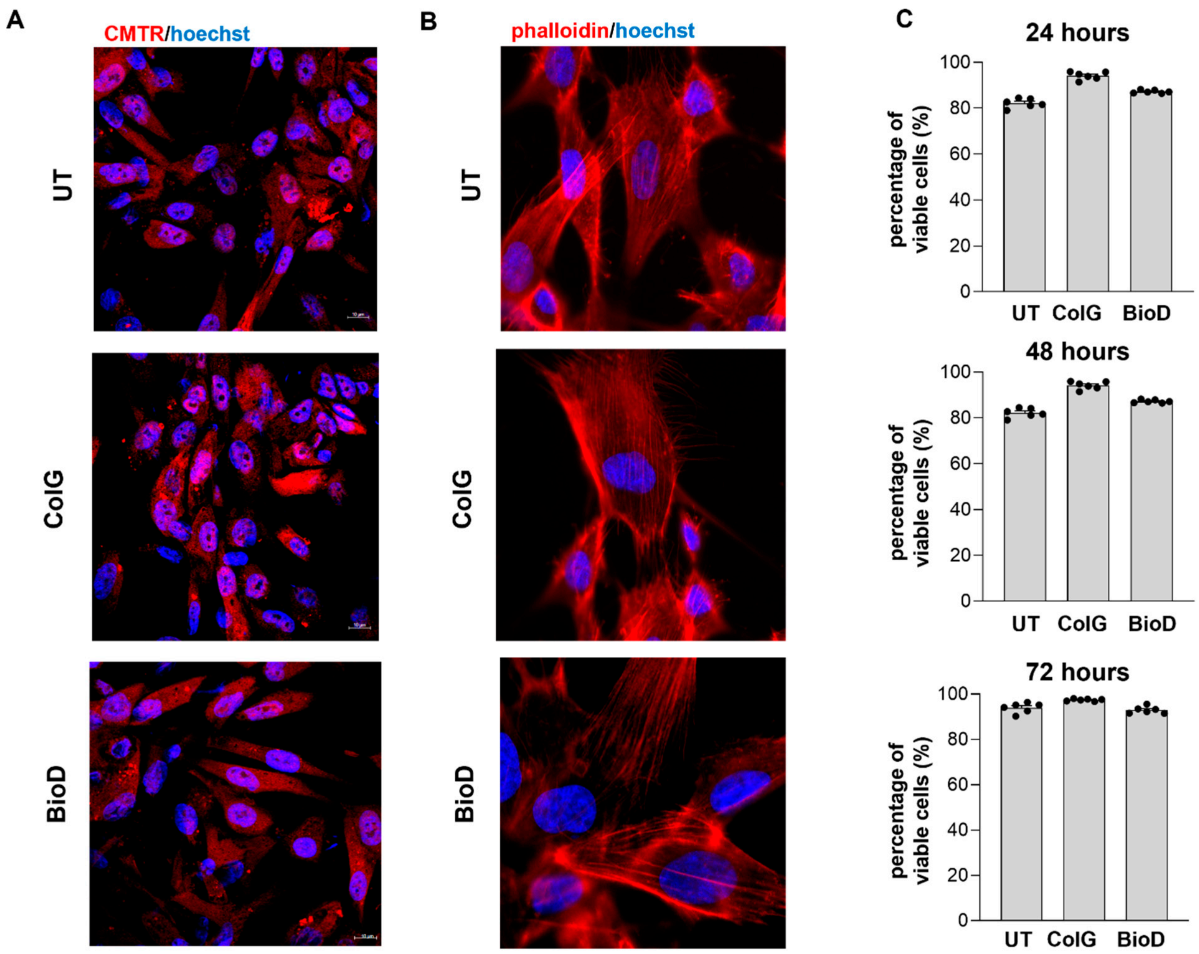

Figure S1. BioD does not alter cell morphology and is non-toxic to cells. A, nFB cells were labelled with Cell Tracker Orange CMTMR Dye (5 µM for 45 minutes) and nuclear counter staining was performed with Hoechst (2 µg/ml for 15 minutes). Cells were then imaged using a confocal microscope (63x). **B,** Cells were stained with Phalloidin Alexa Fluor 568 (red), nuclei were counter stained using DAPI (blue). **C**, Cell viability assessment using flow cytometry using BV510 dye (1:1000 v/v dil). Cell viability was assessed following culturing of the cells on BioD/ColG for 24-72 hours; UT = untreated, ColG = collagen, BioD. Data are mean ± SD. (n=4-6).

Figure S2

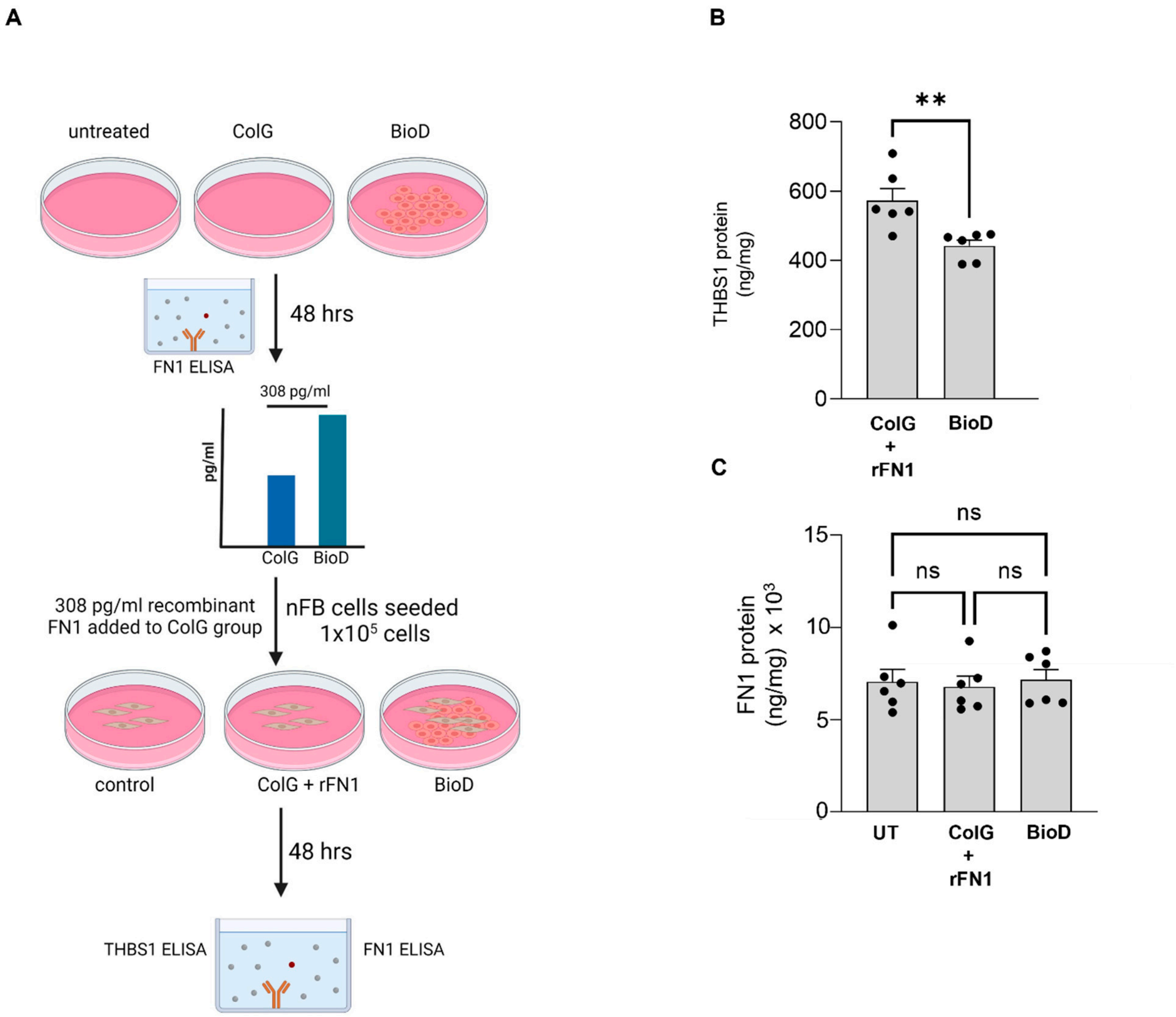

Figure S2. The presence of endogenous FN1 content within BioD is not the cause of THBS1 suppression in nFB cells. To determine of FN1 bound to either BioD influenced its effect on THBS1 or FN1 contents. Culture media was added to BioD or ColG containing cell free culture plates for 48 hours. the cell free CM generated was subjected to FN1 ELISA to determine the levels. The measured amounts were 556.5 pg/ml and 248.5 pg/ml, respectively in BioD or ColG cell free CM. Recombinant FN1 amount (308 pm/ml) that was excess in BioD group was added to ColG CM followed by treatment to Naïve nFB followed by determination of THBS1 and FN1 levels. **A,** Schema of the experimental design; **B-C,** THBS1 and FN1 protein levels; Data are mean ± SD; * $p<0.05$; ** $p<0.01$, *** $p<0.001$ & **** $p<0.0001$ (n=6).

Table S1: RT2 profiler gene list for human extracellular matrix (ECM) and adhesion molecules

Sl. No.	Symbol	Description	Refseq	Fold regulation	p-values (compared to control group)
1	*ADAMTS1*	ADAM metallopeptidase with thrombospondin type 1 motif, 1	NM_006988	-1.03	0.838305
2	*ADAMTS13*	ADAM metallopeptidase with thrombospondin type 1 motif, 13	NM_139025	1.14	0.845554
3	*ADAMTS8*	ADAM metallopeptidase with thrombospondin type 1 motif, 8	NM_007037	1.6	0.548796
4	*CD44*	CD44 molecule (Indian blood group)	NM_000610	-1.28	0.148483
5	*CDH1*	Cadherin 1, type 1, E-cadherin (epithelial)	NM_004360	-2.45	0.394583
6	*CLEC3B*	C-type lectin domain family 3, member B	NM_003278	-1.59	0.056824
7	*CNTN1*	Contactin 1	NM_001843	1.23	0.543424
8	*COL11A1*	Collagen, type XI, alpha 1	NM_080629	1.12	0.687071
9	*COL12A1*	Collagen, type XII, alpha 1	NM_004370	-1.36	0.242472
10	*COL14A1*	Collagen, type XIV, alpha 1	NM_021110	1.41	0.384684
11	*COL15A1*	Collagen, type XV, alpha 1	NM_001855	1.26	0.617649
12	*COL16A1*	Collagen, type XVI, alpha 1	NM_001856	-1.29	0.136240
13	*COL1A1*	Collagen, type I, alpha 1	NM_000088	-2.46	0.000080
14	*COL4A2*	Collagen, type IV, alpha 2	NM_001846	-1.84	0.008924
15	*COL5A1*	Collagen, type V, alpha 1	NM_000093	-2.14	0.002881
16	*COL6A1*	Collagen, type VI, alpha 1	NM_001848	-1.97	0.001457
17	*COL6A2*	Collagen, type VI, alpha 2	NM_001849	-1.75	0.023426
18	*COL7A1*	Collagen, type VII, alpha 1	NM_000094	-2.02	0.011329
19	*COL8A1*	Collagen, type VIII, alpha 1	NM_001850	1.32	0.604779
20	*CTGF*	Cellular communication network factor 2	NM_001901	1.37	0.119101
21	*CTNNA1*	Catenin (cadherin-associated protein), alpha 1, 102kDa	NM_001903	-1.3	0.194433
22	*CTNNB1*	Catenin (cadherin-associated protein), beta 1, 88kDa	NM_001904	1.05	0.751684
23	*CTNND1*	Catenin (cadherin-associated protein), delta 1	NM_001331	1.04	0.997778
24	*CTNND2*	Catenin (cadherin-associated protein), delta 2	NM_001332	1.95	0.573609
25	*ECM1*	Extracellular matrix protein 1	NM_004425	-1.2	0.388839
26	*FN1*	Fibronectin 1	NM_002026	-1.79	0.003012

27	*HAS1*	Hyaluronan synthase 1	NM_001523	-1.28	0.524897
28	*ICAM1*	Intercellular adhesion molecule 1	NM_000201	-1.67	0.046776
29	*ITGA1*	Integrin, alpha 1	NM_181501	-1.24	0.337959
30	*ITGA2*	Integrin, alpha 2 (CD49B, alpha 2 subunit of VLA-2 receptor)	NM_002203	1.06	0.731531
31	*ITGA3*	Integrin, alpha 3 (antigen CD49C, alpha 3 subunit of VLA-3 receptor)	NM_002204	-1.24	0.096950
32	*ITGA4*	Integrin, alpha 4 (antigen CD49D, alpha 4 subunit of VLA-4 receptor)	NM_000885	-1.36	0.025279
33	*ITGA5*	Integrin, alpha 5 (fibronectin receptor, alpha polypeptide)	NM_002205	-1.14	0.414546
34	*ITGA6*	Integrin, alpha 6	NM_000210	1.01	0.929956
35	*ITGA7*	Integrin, alpha 7	NM_002206	-1.31	0.185321
36	*ITGA8*	Integrin, alpha 8	NM_003638	1.13	0.830672
37	*ITGAL*	Integrin, alpha L (antigen CD11A (p180)	NM_002209	-1.3	0.877050
38	*ITGAM*	Integrin, alpha M (complement component 3 receptor 3 subunit)	NM_000632	-1.07	0.673548
39	*ITGAV*	Integrin, alpha V (vitronectin receptor, alpha polypeptide, antigen CD51)	NM_002210	-1.24	0.313057
40	*ITGB1*	Integrin, beta 1	NM_002211	-1.05	0.734041
41	*ITGB2*	Integrin, beta 2 (complement component 3 receptor 3 and 4 subunit)	NM_000211	1.37	0.595484
42	*ITGB3*	Integrin, beta 3 (platelet glycoprotein IIIa, antigen CD61)	NM_000212	1.07	0.632342
43	*ITGB4*	Integrin, beta 4	NM_000213	-1.21	0.389655
44	*ITGB5*	Integrin, beta 5	NM_002213	-1.67	0.026218
45	*ANOS1*	Kallmann syndrome 1 sequence	NM_000216	1.18	0.640749
46	*LAMA1*	Laminin, alpha 1	NM_005559	-1.24	0.187736
47	*LAMA2*	Laminin, alpha 2	NM_000426	1.48	0.143012
48	*LAMA3*	Laminin, alpha 3	NM_000227	1.4	0.453786
49	*LAMB1*	Laminin, beta 1	NM_002291	-1.34	0.071906
50	*LAMB3*	Laminin, beta 3	NM_000228	-1.16	0.418308
51	*LAMC1*	Laminin, gamma 1 (formerly LAMB2)	NM_002293	-1.21	0.059687
52	*MMP1*	Matrix metallopeptidase 1 (interstitial collagenase)	NM_002421	1.32	0.295497
53	*MMP10*	Matrix metallopeptidase 10 (stromelysin 2)	NM_002425	2.39	0.121125
54	*MMP11*	Matrix metallopeptidase 11 (stromelysin 3)	NM_005940	-1.55	0.908427
55	*MMP12*	Matrix metallopeptidase 12 (macrophage elastase)	NM_002426	2.37	0.023004

56	*MMP13*	Matrix metallopeptidase 13 (collagenase 3)	NM_002427	-1.15	0.783767
57	*MMP14*	Matrix metallopeptidase 14 (membrane-inserted)	NM_004995	-1.79	0.096681
58	*MMP15*	Matrix metallopeptidase 15 (membrane-inserted)	NM_002428	-1.01	0.782692
59	*MMP16*	Matrix metallopeptidase 16 (membrane-inserted)	NM_005941	1.2	0.261287
60	*MMP2*	Matrix metallopeptidase 2	NM_004530	-1.32	0.183535
61	*MMP3*	Matrix metallopeptidase 3 (stromelysin 1, progelatinase)	NM_002422	2.64	0.017321
62	*MMP7*	Matrix metallopeptidase 7 (matrilysin, uterine)	NM_002423	-1.01	0.710128
63	*MMP8*	Matrix metallopeptidase 8 (neutrophil collagenase)	NM_002424	-2.18	0.073089
64	*MMP9*	Matrix metallopeptidase 9	NM_004994	-1.22	0.826685
65	*NCAM1*	Neural cell adhesion molecule 1	NM_000615	1.49	0.579328
66	*PECAM1*	Platelet/endothelial cell adhesion molecule	NM_000442	1.38	0.922691
67	*SELE*	Selectin E	NM_000450	1.18	0.631020
68	*SELL*	Selectin L	NM_000655	-1.2	0.497631
69	*SELP*	Selectin P (granule membrane protein 140kDa, antigen CD62)	NM_003005	1.58	0.552810
70	*SGCE*	Sarcoglycan, epsilon	NM_003919	1.46	0.034798
71	*SPARC*	Secreted protein, acidic, cysteine-rich (osteonectin)	NM_003118	-1.12	0.609653
72	*SPG7*	Spastic paraplegia 7 (pure and complicated autosomal recessive)	NM_003119	-1.36	0.357800
73	*SPP1*	Secreted phosphoprotein 1	NM_000582	1.13	0.657438
74	*TGFBI*	Transforming growth factor, beta-induced, 68kDa	NM_000358	-1.94	0.010864
75	*THBS1*	Thrombospondin 1	NM_003246	-2.26	0.000588
76	*THBS2*	Thrombospondin 2	NM_003247	-2.18	0.001316
78	*THBS3*	Thrombospondin 3	NM_007112	-1.35	0.304012
79	*TIMP1*	TIMP metallopeptidase inhibitor 1	NM_003254	-1.31	0.027961
80	*TIMP2*	TIMP metallopeptidase inhibitor 2	NM_003255	-1.8	0.014076
81	*TIMP3*	TIMP metallopeptidase inhibitor 3	NM_000362	-1.32	0.097061
82	*TNC*	Tenascin C	NM_002160	-1.06	0.809940
83	*VCAM1*	Vascular cell adhesion molecule 1	NM_001078	1.49	0.579328
84	*VCAN*	Versican	NM_004385	-1.39	0.512827
85	*VTN*	Vitronectin	NM_000638	1.29	0.651644
86	*ACTB*	Actin, beta	NM_001101	-1.28	0.037634
87	*B2M*	Beta-2-microglobulin	NM_004048	-1.14	0.143946

88	*GAPDH*	Glyceraldehyde-3-phosphate dehydrogenase	NM_002046	1.32	0.015931
89	*HPRT1*	Hypoxanthine phosphoribosyltransferase 1	NM_000194	1.14	0.247049
90	*RPLPO*	L10E/LP0/P0/PRLP0/RPP0	NM_001002	-1.03	0.617374
